# Perceived HIV-Associated Stigma among HIV-Seropositive Men: Psychometric Study of HIV Stigma Scale

**DOI:** 10.3389/fpubh.2015.00171

**Published:** 2015-07-03

**Authors:** Adrian Valle, Ana Cecilia Treviño, Farith Francisco Zambrano, Karla Elizabeth Urriola, Luis Antonio Sánchez, Jesus Eduardo Elizondo

**Affiliations:** ^1^Medical and Health Sciences Program, Department of Basic Sciences, Instituto Tecnológico de Monterrey, Monterrey, Mexico; ^2^Medical and Surgical Dentist Program, Instituto Tecnológico de Monterrey, Monterrey, Mexico; ^3^Doctoral Program in Social Sciences, Instituto Tecnológico de Monterrey, Monterrey, Mexico; ^4^Management and Human Resources, Instituto Tecnológico de Monterrey, Monterrey, Mexico; ^5^Clinical Microbiology and Infectious Diseases, Universidad de Monterrey, San Pedro Garza García, Mexico; ^6^Secretaria de Salud de Nuevo León, Nuevo Leon State Council for AIDS Prevention (COESIDA NL), Monterrey, Mexico; ^7^Doctoral Program in Biotechnology, Biopharmaceuticals and Biopharmaceutical Engineering, Instituto Tecnológico de Monterrey, Monterrey, Mexico; ^8^Doctoral Program in Dentistry, Research in Dentistry, Universitat Internacional de Catalunya, Barcelona, Spain

**Keywords:** HIV, AIDS, social stigma, men who have sex with men, public health, validation studies, psychometrics, Mexico

## Abstract

**Objectives:**

To assess the internal consistency and factor structure of the abridged Spanish version of the Berger HIV Stigma Scale (HSS-21), to provide evidence for its convergent and discriminant validity, and to describe perceived stigma in an urban population from northeast Mexico.

**Methods:**

Seventy-five HIV-positive men who have sex with men (MSM) were recruited. Participants answered the Spanish versions of three Likert-type scales: HSS-21, Robsenberg’s self-esteem scale, and the abbreviated version of the Zung’s Depression Scale.

**Results:**

HSS-21 showed high reliability and validity; its factor structure included four components: concern with public attitudes; negative self-image; disclosure concerns; and enacted stigma. The level of stigma was high in 27 out of 75 (36%) participants; nevertheless, the score found in the component related to disclosure concerns indicated high level of stigma in 68% of participants. The score of HSS-21 was positively correlated with the score of depression and negatively correlated with the score of self-esteem.

**Conclusion:**

Results demonstrated high reliability for the HSS-21; correlations with other scales supported its validity. This scale demonstrated to be a practical tool for assessing stigma among Mexican HIV-positive MSM. High level of stigma was found only in the factor related to disclosure concerns.

**Policy implications:**

Identifying HIV-associated stigma through a short, reliable, and validated instrument will allow the development of interventions that cope and manage stigma in HIV-positive MSM. HSS-21 distinguishes between different dimensions of stigma and will contribute to a better understanding of this phenomenon.

## Introduction

As the human immunodeficiency virus (HIV) epidemic enters in its fourth decade, it is still regarded as an important public health issue worldwide. In turn, the social perception toward those individuals who live with HIV and AIDS (PLWH) continues to be a negative one. HIV’s ways of transmission, its implications regarding the more traditional gender roles, and its association in the social imaginary to socially marginalized groups are the cause of stigma and discrimination in various circles (1). Stigma can be conceptualized as a “mark” (attribute) linking an individual with a set of undesirable characteristics (stereotypes) (2, 3). Three main sub-types of stigma have been described: *acted stigma* refers to the actual experience of acts of discrimination. *Perceived stigma* refers to the fear experienced by an individual in the presence of negative attitudes of a society toward a given attribute considered as undesirable, for example, an ethnic group, a disease, some personal behaviors, etc. A third sub-type is *internalized stigma* (*self-stigma)*, a term which includes the negative beliefs, opinions, and feelings that an individual holds toward a given attribute and toward himself/herself as a result of an internalization process of the societies’ opinions and beliefs toward such attribute (4).

The term HIV- and AIDS-related stigma (HARS) makes reference to the negative appraisal that society as a whole give to any person who lives, or is believed to live with HIV and AIDS. In the psychosocial model developed by Berger et al. (5), the term “*perceived stigma”* is conceptualized as the perception of PLWH of social disqualification (incomplete social acceptance or even rejection), denial or limitation of opportunities (e.g., housing and employment offers, access to medical services, etc.), as well as negative changes to their social identity as a result of their HIV serostatus. This stigmatization process is also associated to other factors involved in judgment formation, such as sexual orientation, diversity of sexual partners, and use of illegal substances. This “blame the victim” attitude increases the isolation sensation and the interiorized sense of shame by the HIV infected individual (1).

From a public health perspective, HARS is an important factor that hinders voluntary testing for a timely diagnosis and treatment of HIV infection (1, 6, 7). HARS is also an obstacle for individual’s serodiagnosis disclosure to their sexual partners, thus prolonging sexual risk behaviors and contributing to HIV transmission (6, 8); particularly in socially stigmatized and discriminated groups such as gay men and men who have sex with men (MSM), where social conditions generated by HARS lead MSM to participate in sexual risk behaviors with a higher risk of acquisition and propagation of HIV (9, 10). Thus, it is not a surprise that worldwide gay men and other MSM are 19 times more likely to be living with HIV than the general population (11). The incidence of HIV among MSM is rising in several parts of the world and Mexico is not the exception (11–13).

Likewise, HARS can also lead PLWH to postpone the use of medical public health services (6, 14), to a suboptimal adherence to antiretroviral therapy (15–17), and to a poor overall health (7, 18). Furthermore, HARS is associated with stress, anxiety, depressive symptomatology, and a decreased quality of life (19, 20). Aforementioned, HARS and its multiple consequences have a deep impact on the HIV epidemic development by reinforcing existing social inequalities with serious implications in public health policies.

Several instruments have been developed and validated to evaluate HARS in PLWH (5, 21–23). One of the best known is the HIV Stigma Scale (HSS-40) developed by Berger et al. (5). This scale has been used to assess perceived stigma among adult PLWH (24–27), likewise it has been adapted for PLWH children (28), and as well to assess perceived stigma of other diseases (29).

The HSS-40 scale assesses how PLWH feel in regards to four dimensions of stigma: (a) personalized stigma (or acted stigma), (b) individuals’ concerns related to disclose his/her own HIV serostatus, (c) negative self-image (self-stigma), and (d) individuals’ concerns related to public attitudes toward PLWH. The instrument has shown evidence of construct validity and high internal consistency for a set of 40 items (α = 0.96), as well as for its factors (α = 0.90–0.93) (20). The HSS-40 Spanish language translation, adaptation, and validation were done by Franke et al. (30), who also developed a shorter version (HSS-21). Likewise, as the complete scale version, the HHS-21 also showed evidence of construct validity (α = 0.84 as a total score), and an adequate-to-high internal consistency (α = 0.68–0.80) in assessing the four dimensions of stigma.

Since HARS not only represents one of the greatest obstacles in preventing new HIV infections but also deeply and negatively affects multiple aspects of HIV treatment, as well as the physical, mental, and emotional well-being of the PLWH (31), it becomes important to assess perceived stigma among PLWH. Considering the lack of adapted and validated instruments for HARS assessment that could improve current HIV and AIDS public health policies in México (e.g., Healthy Border 2010/2010 initiative), this study aims to evaluate the internal consistency, factorial structure, convergent, and discriminant validity of the HSS-21, as well as to describe HARS in a sample of HIV-positive MSM in Nuevo León (Mexican border state with U.S. Texas state).

## Materials and Methods

### Participants

Research involved 185 MSM and who collaborated with non-governmental and community-based organizations members of Nuevo Leon’s Multisectoral STI/HIV/AIDS Response Board (MEMUREVIH). Inclusion criteria involved only male subjects who had had sexual experiences with other men, HIV-positive, aged 18 years or more, and who resided in Nuevo León, Mexican state. Taken into account that most HIV-positive MSM do not disclose their serological HIV status, this study adopted a non-probability sampling and a respondent-driven sampling. The sample consisted of 75 MSM who voluntarily declared to have an HIV-positive serodiagnosis, and who responded to a written or an online survey.

### Recruitment

The data collection process lasted 60 days. Prior to the data collection process, collaboration was requested to the different non-governmental and community-based organizations members of MEMUREVIH to implement the survey in their office location and to their MSM affiliates. Four properly qualified surveyors visited all sites and applied the written or online surveys on the previously agreed dates and time with the aforementioned organizations.

### Procedure

In order to guarantee information input reliability, a digital survey database was created and submitted to a double-quality control check. All subjects were given both verbal and written information about the nature of the study, prior to responding to either the written or online questionnaire. The study procedures were undertaken according to ethical principles with the understanding and written consent of each subject. The study protocol was reviewed and approved by the ethics and research committee at the Tecnologico de Monterrey. Anonymity was guaranteed throughout the process, as well as confidentiality of both physical and electronic data. To access the *online* questionnaire, respondents accessed the survey via the website – (www.encuestas.no-ip.org), and none of the log-in data (IP address, etc.) was stored in the electronic access. Completion of either the written or online survey took an average of 25 min.

### Instrument

The study used an analytical-type structured questionnaire (closed-ended questions and multiple response alternatives) in either written or online formats. The questionnaire explored socio-demographic characteristics, perception of HARS, self-esteem, and depression by means of the following Likert-type scales:

#### HIV Stigma Scale (HSS-21)

The abridged and Spanish-adapted version of the HIV stigma scale (HSS-21) used in this study was developed by Franke et al. (29); it is composed of 21 positively keyed items that are evaluated along a 4-point Likert-type scale (from 1 = strongly disagree to 7 = strongly agree), and was shown to be internally consistent. The sum of these items yields a total score, with higher scores representing a greater degree of perception of stigma.

#### Rosenberg Self-Esteem Scale (RSS-10)

It is composed of 10 items rated on a 4-point Likert-type scale. Possible scores range from 10 to 40, with higher ones indicating greater self-esteem. Among men, scores lower than 28 indicate low self-esteem (32).

#### Zung Depression Scale (ZDS-10)

Zung depression scale constituted of 20 items that involve affective, psychological, and somatic symptoms. Respondents indicate the frequency with which any symptom is experienced: the higher the score, the greater the degree of depressive symptoms. Diaz et al. (33) developed and validated a short, Spanish-adapted version of this scale (ZDS-10); the cut-off point to distinguish the presence of clinically significant depressive symptoms is 22 (sensitivity = 92.3%, specificity = 71.4%).

### Statistical analysis

Two psychometric properties of the items in HSS-21 were estimated: discrimination and internal consistency. Discrimination was estimated by the ability to differentiate in a statistically significant manner the high score group (percentile equal or >73) and the low score group (percentile equal or <27) in the sum score of the 21 items. A mean difference higher than two was considered to be significant, as well as a reliable discriminant property for each one of the assessed items. The internal consistency of the items was estimated by means of: corrected correlation, estimation of Cronbach’s alpha excluding the item, and communalities. It was sought that the corrected correlation values were >0.30, that a non-decrease value of Cronbach’s coefficient alpha after eliminating the item could demonstrated a reliable internal consistency, and that the communalities values were >0.20. Consistency was interpreted as high when it expressed a value equal or >0.70. The distribution fitting to a normal curve was contrasted using Kolmogorov–Smirnov test or Shapiro–Wilk test (34). All data were analyzed with SPSS^®^ software (version 20.0).

The dimensional structure was studied by means of exploratory factor analysis; considering appropriate factoring characteristics: (a) that the determinant of the correlation matrix would tend to 0 (|R| < 0.01), (b) *KMO* > 0.6, and (c) that the null hypothesis of equivalence of the correlation matrix to an identity matrix by Bartlett’s sphericity test would be rejected (34). The extraction was executed by generalized least squares (GLS) and the Promax rotation method of the factor matrix, adjusting the solution to four factors in agreement with the structure found in other studies (5, 30).

The correlations between factors were calculated by Pearson’s correlation coefficient. The correlations between the HSS-21 scores and the EDZ-10 and RSS-10 scores were established by Spearman’s *rho*. The significance of the score difference in the scales ZDS-10 and RSS-10 among the respondents who expressed or did not expressed HARS was carried out by the Mann–Whitney *U* test. The effect size was calculated by Cohen’s d (34).

## Results

### Respondents

The 75 HIV-positive MSM respondents had an average age of 35 (SD = 7.38) years old. Median time since HIV infection diagnosis was 18 months. Average years in school was 13.93 (SD = 3.73) years. At the time of the survey 81.9% of men were working full time, while 10.6% worked part time, and 7.4% were unemployed. Moreover, despite of employment status, 69.2% indicated that they received a monthly income of ≤15,000 MXN ($ 1.146/867.45 €). Regarding sexual orientation, 88% of participants defined themselves as homosexual, 9.23% as bisexual, and 2.67% as heterosexual.

### HSS-21 descriptive statistics

The HSS-21 mean total score was 76.28 (SD = 21.522) and its distribution was fitted to a normal curve (ZK-S = 0.67, *p* = 0.20). Considering the whole scale items, its mean can be transformed into a continued value between 1 and 7 or equal to 3.63. Using the same procedure, most items means scores were also between 2.5 and 4. However, eight items (V2, V6, V11, V12, V14, V15, and V17) had a mean value ≥4 (Table 1).

**Table 1:** **HSS-21 items’ descriptive statistics, interpretation, homoscedasticity, discrimination, and internal consistency**.

Item	Descriptive statistics						K–S		*I*	Homoscedasticity		Discrimination				Consistency		
	*M*	Mdn	Mo	SD	Sk	*K*	Z	*p*		*F*	*p*	MD	*t*	df	*p*	*r*_i, t−i_	α_t−i_	hi
V1	2.95	3	1	2.18	0.63	−1.03	0.29	0.00	2	1.67	0.20	2.7	−4.75	38	0.00	0.48	0.88	0.62
V2	4.73	5	5	1.84	−0.69	−0.17	0.30	0.00	3	18.10	0.00	2.5	−4.54	26.66	0.00	0.52	0.87	0.70
V3	3.85	3	3	2.23	0.16	−1.33	0.21	0.00	2	0.79	0.38	2.6	−3.86	38.00	0.00	0.38	0.88	0.70
V4	2.73	3	1	1.95	0.81	−0.47	0.28	0.00	2	2.10	0.15	2.2	−4.41	38.00	0.00	0.36	0.88	0.44
V5	3.21	3	1^a^	1.96	0.14	−1.36	0.25	0.00	2	0.05	0.82	2.4	−4.22	38.00	0.00	0.43	0.88	0.56
V6	4.07	5	5	1.65	−0.55	−0.39	0.33	0.00	3	3.63	0.06	2.6	−5.81	38.00	0.00	0.55	0.87	0.74
V7	2.52	1	1	1.83	0.83	−0.50	0.32	0.00	2	9.62	0.00	2.6	−5.64	30.76	0.00	0.56	0.87	0.71
V8	2.20	1	1	1.68	1.16	0.28	0.36	0.00	1	12.10	0.00	2.5	−5.54	29.67	0.00	0.55	0.87	0.62
V9	3.53	3	5	1.72	−0.16	−9.98	0.26	0.00	2	0.21	0.65	2.3	−5.04	38.00	0.00	0.40	0.88	0.64
V10	1.99	1	1	1.48	1.35	0.97	0.39	0.00	1	20.14	0.00	2.3	−5.68	23.89	0.00	0.56	0.87	0.68
V11	4.44	5	5	1.70	−0.52	−0.16	0.31	0.00	3	8.37	0.00	2.5	−5.15	32.44	0.00	0.55	0.87	0.73
V12	6.31	7	7	1.21	−1.94	4.45	0.42	0.00	4	3.35	0.07	0.5	−1.12	38.00	0.27	0.06	0.88	0.65
V13	3.00	3	1	2.00	0.67	−0.23	0.23	0.00	2	15.31	0.00	2.5	−4.86	26.32	0.00	0.41	0.88	0.60
V14	4.44	5	5	1.56	−0.50	0.55	0.32	0.00	3	0.02	0.90	2.7	−6.76	38.00	0.00	0.58	0.87	0.59
V15	4.23	5	7	2.39	−0.28	−1.48	0.21	0.00	3	9.30	0.00	4.4	−8.63	25.59	0.00	0.68	0.87	0.76
V16	2.16	5	5^a^	2.16	0.33	−1.12	0.21	0.00	1	6.81	0.01	2.7	−4.34	32.67	0.00	0.44	0.88	0.64
V17	4.84	5	7	2.13	−0.53	−0.99	0.23	0.00	3	6.73	0.01	3.3	−5.96	28.57	0.00	0.63	0.87	0.67
V18	3.87	3	3	2.07	0.44	−0.95	0.23	0.00	2	8.25	0.00	2.2	−3.47	31.76	0.00	0.32	0.88	0.77
V19	2.97	3	3	1.72	0.55	−0.36	0.24	0.00	2	2.25	0.14	2.1	−4.58	38.00	0.00	0.38	0.88	0.74
V20	3.37	3	1	2.02	0.26	−1.10	0.20	0.00	2	6.75	0.01	2.9	−5.22	29.50	0.00	0.53	0.87	0.62
V21	3.03	3	3	1.82	0.64	−0.30	0.25	0.00	2	9.76	0.00	3.1	−6.94	27.84	0.00	0.64	0.87	0.73

**M*, mean; Mdn, median; Mo, mode; SD, standard deviation; Sk, skewness; *K*, kurtosis; K–S, Kolmogorov–Smirnov test; *p*, probability; I, interpretation; 1, totally in disagreement; 2, in disagreement; 3, in agreement; 4, totally in agreement; MD, mean difference between the group with high scores and the group with low scores in the scale; df, degrees of freedom; *r*_i,t-i_, correlation between the item and the scale; α_t−i_, Cronbach’s alpha coefficient after removing the item; hi, communality*.

*^a^α_t−i_, Cronbach’s alpha coefficient after removing the item (significance α ≥ 0.70)*.

### Discrimination, internal consistency, and validity of HSS-21 items

HSS-21 presented a high internal consistency (α = 0.88). Items expressed reliable internal consistency values, since deletion of none of them increased Cronbach’s alpha coefficient. Items’ corrected correlations ranged from 0.32 to 0.68 (except item V12, whose corrected correlation was 0.06). Initial communalities varied between 0.44 and 0.77, except for item V12 (MD = 0.05). Thus, with the exception of item V12, the remaining 20 items had reliable psychometric properties (Table 1).

### HSS-21 dimensional structure

The correlation matrix of the 21 items of the HSS-21 scale showed adequate properties for factor extraction. Its determinant tended to 0 (|R| < 0.01), the *KMO* index was >0.6 (*KMO* = 0.777), and the null hypothesis of equivalence of the correlation matrix to an identity matrix by Bartlett’s sphericity test was rejected [χ^2^ (210, *N* = 75) = 752.413, *p* < 0.001]. Four components were extracted, which accounted for 60.51% of the total variance.

After rotating the factor component matrix by the Promax method and performing the extraction by GLS, four factors were found with a high internal consistency; they were constituted by positive indicators and factor loadings >0.32 (Table 2). The first factor (self-stigma, VNS) integrated by six items (V1, V4, V7, V8, V10, and V16) related to feelings of not being as good as others and emotion of guilt or shame (α = 0.81). The second factor (acted stigma, VES) integrated by five items (items V13, V18, V19, V20, and V21) related to the consequences of others finding out the individuals’ serodiagnosis and personal experiences of rejection (α = 0.83). The third factor (concern about public attitude toward HIV and AIDS, VPA) integrated by five items (V5, V6, V9, V11, and V14) related to what PLWH believe that other people could think of them if they knew their serostatus (α = 0.83). The fourth factor (PLWH concern about HIV serodiagnosis disclosure, VDC) integrated by five items (V2, V3, V12, V15, and V17) related to the perceived need of controlling HIV serodiagnosis information (α = 0.76). Item 21 had a high factor loading in two factors. Nevertheless, including it in the second factor increased its internal consistency from 0.80 to 0.83, due to its content item 21 is highly related to acted stigma. The correlations between factors were medium (*r* = 0.32–0.46), with the exception of the correlation between factors 3 and 4, which was small (*r* = 0.21).

**Table 2:** **HSS-21 factors’ loadings from exploratory factor analysis**.

Item		Factor			
		1	2	3	4
V6	Most people believe a person who has HIV is dirty	0.88			
V11	Most with HIV are rejected when others learn I have HIV	0.77			
V9	Most people think a person with HIV is disgusting	0.73			
V5	People with HIV are treated like despicable persons	0.71			
V14	Most people are uncomfortable around someone with HIV	0.59			
V21	People seem afraid of me because I have HIV	0.53			0.44
V7	Having HIV makes me feel unclean		0.93		
V8	I feel set apart, isolated from the rest of the world		0.71		
V10	Having HIV makes me feel I am a bad person		0.68		
V1	I feel guilty because I have HIV		0.67		
V4	I feel I am not as good as others because I have HIV		0.36		
V16	Having HIV in my body is disgusting to me		0.32		
V2	Telling someone I have HIV is risky			0.77	
V15	I worry that people may judge me when they learn I have HIV			0.76	
V17	I worry people who know I have HIV will tell others			0.69	
V3	I work hard to keep my HIV a secret			0.65	
V12	I am very careful whom I tell that I have HIV		−0.39	0.50	
V18	I regret having told some people that I have HIV				0.93
V19	As a rule, telling others has been a mistake				0.92
V20	Some people act as though it is my fault I have HIV				0.51
V13	Some people who know have grown more distant	0.37			0.48

*Extraction method: GSL. Rotation method: Promax with Kaiser normalization*.

### HSS-21 factors’ descriptive statistics

The score range for all items is from 1 to 7 and the higher score results are correlated to a high stigma. Considering the number of items in each of the four factors, their mean can be transformed into a continuous value within 1 and 7. Such value can be interpreted as the answer label for each item, dividing the answer range value by four intervals of constant amplitude. Table 3 exposes factor descriptive statistics.

**Table 3:** **HSS-21 factors’ descriptive statistics**.

Factor	Descriptive statistics						K–S		Interpretation
	*M*	Mdn	Mo	SD	Sk	*K*	*Z*	*p*	
VNS	3.99	3.83	3.83	1.22	−0.17	−0.66	0.10	0.07	In disagreement
VES	3.15	3	1	1.48	0.56	−0.40	0.10	0.07	In disagreement
VPA	3.94	4.2	5	1.33	−0.22	−0.71	0.18	0.01	In disagreement
VDC	4.79	4.6	4.6	1.46	0.81	−0.47	0.10	0.07	In agreement

**M*, mean; Mdn, median; Mo, Mode; SD, standard deviation; Sk, skewness; *K*, kurtosis; K–S, Kolmogorov–Smirnov test; *p*, probability; VNS, negative self-image; VES, enacted stigma; VPA, public attitude; VDC, disclosure concerns*.

The mean of VNS can be interpreted as a non-definitive disagreement with HARS dimension. Means comparison among the 36 (48%) respondents who expressed stigma [*M* = 5.04, SD = 0.59; 95% IC = (4.85, 5.24)] and the 39 (52%) respondents who did not express stigma [*M* = 3.02, SD = 0.74; 95% IC = (2.78, 3.26)] were statistically significant (*p* < 0.01).

Also, VES mean can be interpreted as a non-definitive disagreement with HARS dimension. The comparison of means among the 23 (30.7%) respondents who expressed stigma [*M* = 4.96, DE = 0.69; 95% IC = (4.66, 5.26)] and the 52 (69.3%) respondents who did not express stigma [*M* = 2.34, SD = 0.91; 95% IC = (2.09, 2.60)] was statistically significant (*p* < 0.01).

The VPA mean can be interpreted as a non-definitive disagreement with HARS dimension. The comparison of means among the 40 (53.3%) respondents who expressed stigma [*M* = 4.99, DE = 0.65; 95% IC = (4.78, 5.20)] and the 35 (36.7%) respondents who did not express stigma [*M* = 2.74, SD = 0.75; 95% IC = (2.48, 3.00)] was statistically significant (*p* < 0.01).

Likewise, VDC mean can be interpreted as a non-definitive disagreement with HARS dimension. Means comparison among the 51 (68%) respondents who expressed stigma [*M* = 5.59, DE = 0.94; 95% IC = (5.32, 5.85)] and the 24 (32%) respondents who did not express stigma [*M* = 3.10, SD = 0.74; 95% IC = (2.79, 3.41)] were statistically significant (*p* < 0.01).

### ZDS-10 descriptive statistics

ZDS-10 scale had an average score of 20.48 (SD = 4.82) with a non-normal distribution curve (ZK-S = 0.11, *p* = 0.02). Likewise, scores among the 48 respondents who did not express stigma showed a non-normal distribution curve (ZS-W = 0.93, *p* = 0.01). Whereas, distribution among the 27 respondents who expressed stigma was adjusted to a normal curve (ZS-W = 0.95, *p* = 0.17).

After running the Mann–Whitney *U* test, it was found that the respondents with HARS had significantly higher scores in ZDS-10 [*M* = 22.41, SD = 4.40, *n* = 27; 95% IC (20.67, 24.15)] than the scores of the respondents without HARS [*M* = 19.40, SD = 4.74, *n* = 48; 95% IC (18.02, 20.77)], with a medium size effect of stigma on the total ZDS-10 score; *Z* = −2.37, *p* < 0.02, *d* = 0.65, 95% IC (0.16, 1.13).

Of the 48 HIV-positive MSM respondents who did not express HARS, 14 (29.17%) presented clinically significant symptoms of depression, while 34 (70.83%) did not show such symptoms. Of the 27 respondents who expressed HARS, 16 (59.26%) showed clinically significant symptoms of depression, while 11 (40.74%) did not showed such symptoms.

### RSS-10 descriptive statistics

The mean in the total score of the RSS-10 was 34.43 (SD = 4.78) with a non-normal distribution curve (ZK-S = 0.20, *p* < 0.0.001). Similarly, the distribution of the scores between the respondents who did not express stigma (ZS-W = 0.87, *p* = 0.001) and those who did express stigma (ZS-W = 0.88, *p* = 0.007) presented a non-normal distribution curve.

After running the Mann–Whitney *U* test, it was found that the HIV-positive MSM respondents who did not express HARS had significantly higher RSS-10 scores [*M* = 35.53, SD = 3.62, 95% IC (34.50, 36.60)] than those of the respondents who did express HARS [*M* = 32.48, SD = 5.93, 95% IC (30.14, 34.83)], with a medium size effect of stigma on the total RSS-10 score; *Z* = −2.26, *p* < 0.03, *d* = −0–67, 95% IC (−1.14, −0.18).

Of the 48 respondents who did not express HARS, one (2%) respondent presented a low self-esteem and 47 (98%) presented a high self-esteem. Of the 27 respondents who expresses HARS, six (22.2%) respondents presented a low self-esteem and 16 (77.8%) presented a high self-esteem.

### Validity evidences

The total HSS-21 score expressed convergent validity with a significant and direct correlation to the ZDS-10 total score. The factors that expressed a high correlation were VNS (ρ = 0.336, *p* < 0.001) and VES (ρ = 0.252, *p* < 0.01). Also, the total HSS-21 score demonstrated discriminant validity with significant and reverse correlation to RSS-10 total score. HSS-21 factors showed correlations from small (VNS, VDC, and VES) to trivial (VPA) with the RSS-10, but did not reached a statistical significance.

## Discussion

Stigma can be conceptualized as a deeply degrading attribute that binds an individual with one or more undesirable characteristics and finally diminish socially his/her self-image. Concern about stigma is widely extended among PLWH, although this is not universal (35). The results of the study determined that the Spanish-adapted version of the HSS-21 scale developed by Franke et al. (30) has good overall internal consistency, reliability, and psychometric characteristics among the sub-population studied (HIV-positive MSM).

From the HSS-21 total scores, more than half (64%) of the HIV-positive MSM respondents did not express HARS. However, VDC factor scores showed that 68% of the respondents perceived HARS. The HSS-21 score had a significant and direct correlation with the ZDS-10 total score, showing evidence of convergent validity. Likewise, it also presented a significant and reverse correlation with the RSS-10 total score, demonstrating evidence of discriminant validity. The respondents who perceive HARS had significantly higher scores (*p* < 0.02) in ZDS-10 and significantly lower scores (*p* < 0.03) in RSS-10 than the respondents who do no perceive HARS.

There are only a small number of published studies on interventions and programs designed to reduce HARS. Given the difficulties in defining and measuring stigma, few such interventions and programs described in the literature have been rigorously evaluated in developing countries (10, 21). One of the limitations of this study – typically found in other HIV-related studies – is the fact that it was conducted with a small and convenience sample. For this reason, the study does not offer sufficient data to assess the true meaning of the stigma scores of the scale, and herein reported results and derived conclusions should be considered as hypothetical for similar social groups, which limits the ability to apply it to the general PLWH population, or to draw conclusions about the impact of perceptions of HARS in Mexico. Moreover, HARS data were obtained through a self-reported evaluation and therefore they can be different from those obtained through an interview, projective tests, or psychophysiological tests, etc. Also, sexual orientation, gender identity, social, economic, and cultural differences need to be assessed. Nevertheless, the findings can serve as the basis for HARS measurement research with Spanish-speaking populations in Mexico and other countries.

To improve our understanding of HARS, it is necessary to focus on a series of distinguishable variables across different societies. Implementing HARS evaluation in PLWH is important, as it can lead to identify clinically significant symptoms of depression which could have a negative influence in treatment adherence (15–17), it could also limit the use of public health services (36, 37) and limit social support seeking due to fear of rejection (38, 39). Identifying perceived stigma among PLWH through a short, reliable, and validated instrument provides data that will help governments make efficient and effective use of resources spent on stigma and discrimination reduction as well as the opportunity to develop public health interventions that help PLWH to cope and manage stigma. The implementation and evaluation of these strategies will benefit from the availability of a widely validated and user-friendly psychometric instruments to assess the HARS.

This study was the first to use the Spanish-adapted version of the HSS-21 scale in HIV-positive MSM in Mexico, and the findings help move from evaluating HARS to implementing reduction interventions to mitigate the impact of HARS and promote empowerment among HIV-positive men and to enhance the sexual health of gay and bisexual men through a community-based process involving the MEMUREVIH (program developed by a consortium of members who represent four sectors: academia, government, key population, non-governmental and community-based organizations in Nuevo León state). Through study results, the MEMUREVIH intervention aimed to diminish HARS, create greater support for HIV-positive men, make disclosure safer and easier, discourage reliance on disclosure to prevent HIV transmission and encourage testing. The contact hypothesis suggests that facilitating social interaction between PLWH and others (e.g., healthcare providers, friends, family, co-workers, etc.) may help to reduce prejudice, stereotypes, and discrimination among others via the mechanisms of increased knowledge of HIV, reduced social anxiety regarding PLWH, and increased empathy toward PLWH (40). The corresponding author is currently working on MEMUREVIH intervention data.

As HARS places people at a substantial social disadvantage, it increases their exposure to risks and limits access to protective factors, potentially adding to their burden of disease or disability. Stigma not only predisposes PLWH to greater HARS and discrimination but also critically reinforces stereotyping and status loss of all them, regardless of how they may have acquired the infection. In such manner, underestimating the insidious power of stigma jeopardizes the success of public health programs aimed to prevent, timely detect, and treat HIV and AIDS. HSS-21 scale distinguishes different dimensions of stigma and contributes to better understand HARS impact in public health-related issues. Furthermore, the intention is also to encourage researchers to consider additional ways to reduce stigma and discrimination, acknowledging and addressing HARS challenges with research methodology; thereby, creating a sense of timeliness and urgency for the development and testing of HARS, and thus, contributing with discrimination reduction efforts. It is only with stronger, more nuanced understandings of HARS that we will be able to break the associations between HIV stigma and indicators of poor affective, behavioral, and physical health and well-being among PLWH within interventions.

## Conflict of Interest Statement

The authors declare that the research was conducted in the absence of any commercial or financial relationships that could be construed as a potential conflict of interest.
